# Impact of FAPI-PET/CT on Target Volume Definition in Radiation Therapy of Locally Recurrent Pancreatic Cancer

**DOI:** 10.3390/cancers13040796

**Published:** 2021-02-14

**Authors:** Jakob Liermann, Mustafa Syed, Edgar Ben-Josef, Kai Schubert, Ingmar Schlampp, Simon David Sprengel, Jonas Ristau, Fabian Weykamp, Manuel Röhrich, Stefan A. Koerber, Uwe Haberkorn, Juergen Debus, Klaus Herfarth, Frederik L. Giesel, Patrick Naumann

**Affiliations:** 1 Department of Radiation Oncology, Heidelberg University Hospital, Im Neuenheimer Feld 400, 69120 Heidelberg, Germany; mustafa.syed@med.uni-heidelberg.de (M.S.); Kai.schubert@med.uni-heidelberg.de (K.S.); ingmar.schlampp@med.uni-heidelberg.de (I.S.); simon.sprengel@med.uni-heidelberg.de (S.D.S.); jonas.ristau@med.uni-heidelberg.de (J.R.); fabian.weykamp@med.uni-heidelberg.de (F.W.); stefan.koerber@med.uni-heidelberg.de (S.A.K.); juergen.debus@med.uni-heidelberg.de (J.D.); klaus.herfarth@med.uni-heidelberg.de (K.H.); patrick.naumann@med.uni-heidelberg.de (P.N.); 2 Heidelberg Institute of Radiation Oncology (HIRO), Im Neuenheimer Feld 400, 69120 Heidelberg, Germany; 3 National Center for Tumor Diseases (NCT), Im Neuenheimer Feld 460, 69120 Heidelberg, Germany; 4 Heidelberg Ion-Beam Therapy Center (HIT), Im Neuenheimer Feld 450, 69120 Heidelberg, Germany; 5 Department of Radiation Oncology, University of Pennsylvania, Philadelphia, PA 19104, USA; Edgar.Ben-Josef@pennmedicine.upenn.edu; 6 Department of Nuclear Medicine, Heidelberg University Hospital, Im Neuenheimer Feld 400, 69120 Heidelberg, Germany; manuel.roehrich@med.uni-heidelberg.de (M.R.); uwe.haberkorn@med.uni-heidelberg.de (U.H.); frederik.giesel@med.uni-heidelberg.de (F.L.G.); 7 Clinical Cooperation Unit Radiation Oncology, German Cancer Research Center (DKFZ), Im Neuenheimer Feld 280, 69120 Heidelberg, Germany; 8 German Cancer Consortium (DKTK), Partner Site Heidelberg, German Cancer Research Center (DKFZ), Im Neuenheimer Feld 280, 69120 Heidelberg, Germany

**Keywords:** FAPI, pancreatic cancer, interobserver variability, target definition, cancer-associated fibroblasts

## Abstract

**Simple Summary:**

We demonstrate how manual target definition based on contrast-enhanced computed tomography is highly unreliable and inconsistent. In a second step, we used a novel positron emission tomography tracer, FAPI (68Ga-labeled fibroblast activation protein inhibitor) for target volume definition. FAPI-PET/CT contains biologic information as it visualizes cancer associated fibroblasts. The pioneering use of FAPI PET/CT in radiation treatment planning improved target definition in locally recurrent pancreatic cancer.

**Abstract:**

(1) Background: A new radioactive positron emission tomography (PET) tracer uses inhibitors of fibroblast activation protein (FAPI) to visualize FAP-expressing cancer associated fibroblasts. Significant FAPI-uptake has recently been demonstrated in pancreatic cancer patients. Target volume delineation for radiation therapy still relies on often less precise conventional computed tomography (CT) imaging, especially in locally recurrent pancreatic cancer patients. The need for improvement in precise tumor detection and delineation led us to innovatively use the novel FAPI-PET/CT for radiation treatment planning. (2) Methods: Gross tumor volumes (GTVs) of seven locally recurrent pancreatic cancer cases were contoured by six radiation oncologists. In addition, FAPI-PET/CT was used to automatically delineate tumors. The interobserver variability in target definition was analyzed and FAPI-based automatic GTVs were compared to the manually defined GTVs. (3) Results: Target definition differed significantly between different radiation oncologists with mean dice similarity coefficients (DSCs) between 0.55 and 0.65. There was no significant difference between the volumes of automatic FAPI-GTVs based on the threshold of 2.0 and most of the manually contoured GTVs by radiation oncologists. (4) Conclusion: Due to its high tumor to background contrast, FAPI-PET/CT seems to be a superior imaging modality compared to the current gold standard contrast-enhanced CT in pancreatic cancer. For the first time, we demonstrate how FAPI-PET/CT could facilitate target definition and increases consistency in radiation oncology in pancreatic cancer.

## 1. Introduction

Pancreatic cancer is one of the most lethal cancers with rising incidence rates [1]. In 2020, there were approximately 47,050 pancreatic cancer-related deaths in the United States, representing 7.8% of all national cancer-deaths, according to estimations by the National Cancer Institute. Currently, surgery is the only curative therapy option, but only 20% of all pancreatic cancers are diagnosed in an operable stage of the disease [2]. Most of the patients develop tumor recurrence in form of locally recurrent pancreatic cancer or distant metastases. Up to 30% of all tumor deaths are related to local disease burden [3]. Evidence for the therapeutic management of locally recurrent pancreatic cancer is poor. Treatment should be performed within clinical trials and re-resection should be considered [4]. Radiotherapy or chemoradiation is an alternative option in the treatment of locally recurrent pancreatic cancer especially in inoperable cases; but, the efficacy of radiotherapy remains poor. Retrospective analyses observed a median overall survival of approximately 16 months after radiotherapy [5,6]. To improve survival through radiotherapy in pancreatic cancer, several modern radiation techniques are currently investigated. The use of carbon ion radiotherapy could improve oncological outcomes, as shown for locally recurrent pancreatic cancer patients by a retrospective analysis of Kawashiro et al. [7]. The National Comprehensive Cancer Network (NCCN) guidelines recommend the consideration of stereotactic body radiotherapy (SBRT) in this situation as there are promising data supporting the use of SBRT in pancreatic cancer [8]. 

Modern radiation techniques with high conformity such as particle therapy or stereotactic body radiotherapy (SBRT) allow the delivery of higher radiation doses, precisely to the delineated tumor tissue. Irradiating with such high accuracy requires equally advanced target volume definition in radiotherapy planning to achieve tumor control and less toxicity. However, with contrast-enhanced computed tomography (CT) as the current gold standard for imaging of pancreatic cancer, differentiation of locally recurrent pancreatic cancer is often challenging [9,10]. Although there have been improvements concerning imaging quality in the past decade, tumor tissue can often hardly be differentiated from postoperative fibrosis or other postoperative anatomical changes in locally recurrent pancreatic cancer. In a meta-analysis of seven retrospective studies, CT imaging’s pooled estimated Sensitivity and Specificity were 0.70 and 0.80 and the ones of positron emission tomography with 18F-Fluorodeoxyglucose (FDG-PET)/CT were approximately 0.9 each [11]. Nevertheless, FDG avidity in pancreatic tumors is only marginally higher than that of healthy pancreas parenchyma itself. In addition, false-positive FDG uptake is often seen in peritumoral or post-procedural inflammation and false-negative results are common in hyperglycemic patients or small lesions [12]. To improve target definition, there is an urgent need of higher imaging quality. 

Fibroblast activation protein inhibitor (FAPI)-PET/CT is a novel imaging modality that uses inhibitors of FAP to visualize cancer associated fibroblasts (CAFs). A previous study has already demonstrated high FAPI uptake in pancreatic ductal adenocarcinoma (PDAC), resulting in high-contrast imaging [13]. In addition, the exciting novelty of FAPI imaging lies in the biological information hidden behind its standard uptake values (SUVs). PDAC exhibits dense desmoplasia, mainly consisting of CAFs. CAFs, especially in higher density are known to play an essential role in the development, progression, expansion and therapy resistance of PDACs [14]. Recently, Röhrich et al. could demonstrate that FAPI-PET/CT imaging leads to significant changes in tumor staging compared to standard imaging [15]. In seven of the 19 analyzed patients, FAPI-PET/CT even influenced oncological treatment.

In the present study, in a first step, we aimed to explore the interobserver variability in contouring gross tumor volumes (GTVs) of locally recurrent pancreatic cancer patients by six different radiation oncologists of two different institutions. In a second step, we evaluated the suitability of FAPI-PET/CT imaging as a tool for automated target volume definition and correlated the FAPI-PET/CT-based delineation with the manually contoured GTVs.

## 2. Materials and Methods

### 2.1. CT Imaging and Target Volume Definition by Radiation Oncologists

GTVs were contoured individually by six radiation oncologists from two different institutions. Clinical information was extracted from the charts. GTVs were contoured on an axial native CT scan with a slice thickness of 3 mm. Additionally, arterial, venous, and late-venous contrast-enhanced CT-series were available to improve decision making. The FAPI-PET/CT imaging information was not available to these six radiation oncologists. Accuray Precision^®^ Treatment Planning System (Accuray Incorporated, Sunnyvale, CA, USA) was used for target volume definition.

### 2.2. FAPI-PET/CT Imaging

68Ga-FAPI-04 was synthesized and labeled as previously described [16,17]. PET imaging was performed with a Biograph mCT Flow scanner (Siemens) as previously described [13,18]. Native low-dose CT scans were followed by PET scans performed in 3D-mode (matrix 200 × 200). After correction of the corresponding emission data, reconstructions were generated. For all 7 patients, PET scans were obtained 40–60 min after 68Ga-FAPI-4-injection.

### 2.3. Automated Target Volume Definition in FAPI-PET/CT-Scans

A seventh radiation oncologist, not involved in the conventional GTV contouring, automatically delineated tumors using FAPI-PET/CT. Here, the SUVs of healthy tissues were compared to the tumorous tissue as previously described [19]. Briefly, an individual SUV of the healthy tissue surrounding the tumor was quantified using the region-of-interest method as a first step. The resulting individual background value was used to define three different thresholds of FAPI uptake in the primary tumor (1.5, 2.0, and 2.5). Three different sized GTVs were automatically generated using these FAPI-uptake thresholds. The FAPI-GTVs were correlated with anatomical CT-imaging for plausibility and if needed, corrected for false positive/negative FAPI avidity. 

### 2.4. Image Registration

Rigid image registration of FAPI-PET/CT-scans and conventional planning CT-scans was performed using the software Syngo.via RT Image Suite (Siemens AG, Munich, Germany). Manual adjustments were made in all cases for adequate matching. After successful image registration, FAPI-GTVs were transferred from the PET/CT-scan to the conventional CT-scan for comparison.

### 2.5. Interobserver Variability

Interobserver variability was evaluated by comparison of the delineated GTVs. We hypothesized that there is no gold standard GTV, since target volume definition is a subjective task. Therefore, we compared each radiation oncologist’s GTV with that of the other radiation oncologists using the following established comparison methods. The according geometries were analyzed using the Raystation software (RaySearch Laboratories, Eugeniavagen, Sweden). 

### 2.6. Comparison of Volume Geometries

The dice similarity coefficient (DSC) was defined as previously described [20,21]. A value of 0 represents no overlap and a value of 1 represents a complete overlap of two analyzed volumes. Comparing the GTV of one radiation oncologist to the ones of the other five radiation oncologists, five different DSCs were generated per radiation oncologist per patient. All DSCs of one radiation oncologist were compared to the DSCs of the other radiation oncologists. 

Precision, Sensitivity, and Specificity were defined as preciously described [21]. In Precision and Sensitivity, a value of 0 represents no overlap and a value of 1 represents a complete overlap of two analyzed volumes. In Specificity, a value of 1 represents a complete overlap, otherwise a value less than 1 is observed. 

The mean distance to agreement (mean DTA, in cm) represents the mean of all distances, when each voxel on the surface of one volume is assigned with a minimum distance of the voxel to the surface of the compared volume. The maximum distance to agreement (max DTA, in cm) is defined by taking the maximum of the measured distances. A value of 0 represents a complete overlap. 

Precision, Sensitivity, Specificity, and the distances to agreement were evaluated similar to the DSC.

### 2.7. GTV Size Comparison

Volumes in ccm were extracted from the Raystation software (RaySearch Laboratories, Eugeniavagen, Sweden). The sizes of FAPI-GTVs were compared to the radiation oncologists’ conventional GTVs.

### 2.8. Statistics

Volume geometries were compared calculating mean and standard deviations of a confidence interval of 95%. Statistical significances between the different observers were analyzed by one-way analysis of variance (ANOVA). A *p*-value of < 0.05 was considered statistically significant. GTV sizes were compared using the Wilcoxon Rank Sum Test. All statistical analyses were performed with SPSS Statistics (International Business Machines Corporation: IBM, New York, NY, USA).

## 3. Results

### 3.1. Patient and Tumor Characteristics

From 2017 until 2019, we irradiated seven locally recurrent PDAC patients who received FAPI-PET/CT in addition to the conventional CT imaging for radiotherapy planning. All patients were irradiated at our Ion-Beam Therapy Center with carbon ions with a total dose of 48 Gy (RBE) in 12 fractions. Patient characteristics are described in Table 1.

### 3.2. Target Volume Definition by Radiation Oncologists

**Figure 1:**
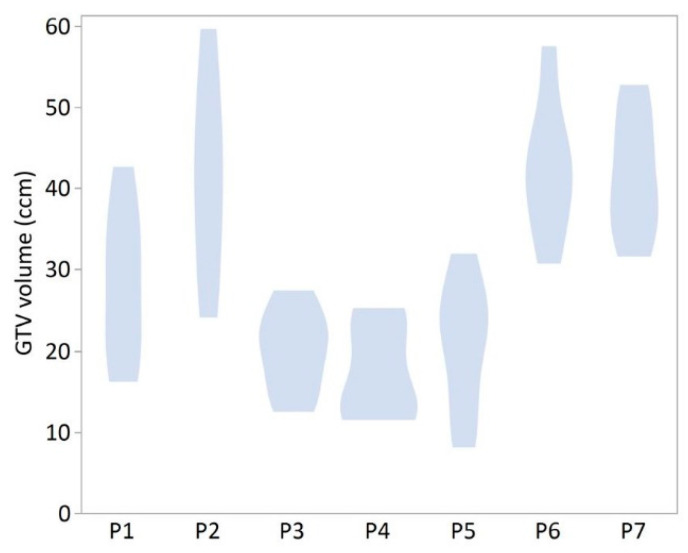
Largely ranging gross tumor volumes (GTVs) per patient, based on manual target definition by six different radiation oncologists. Violin plot of seven different locally recurrent pancreatic cancer patients is shown (P1–P7).

**Figure 2:**
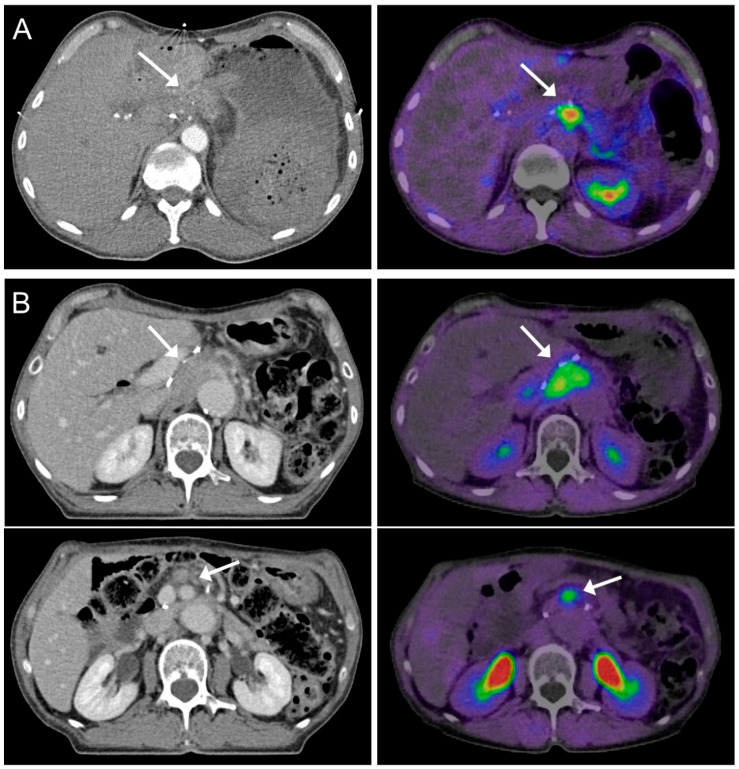
Precision of fibroblast activation protein inhibitor positron emission tomography/computed tomography (FAPI-PET/CT) compared to CT. (**A**) Imaging of a 77-year old patient suffering from locally recurrent pancreatic ductal adenocarcinoma (PDAC). Based on CT imaging on the left, the tumor (white arrow) can hardly be differentiated from the surrounding tissue. It can be located relatively precisely in the fused slice of the according FAPI-PET/CT scan on the right. (**B**) Imaging of a 71-year old nodal positive locally recurrent PDAC patient. The main tumor (white arrow) can be defined adequately in CT imaging as well as in FAPI-PET/CT imaging (upper right). In the lower left, the white arrow marks a lymph node that can easily be overlooked without FAPI-PET/CT information (lower right). In the present study, none of the six radiation oncologists contoured this lymph node as part of the gross tumor volume (GTV).

**Figure 3:**
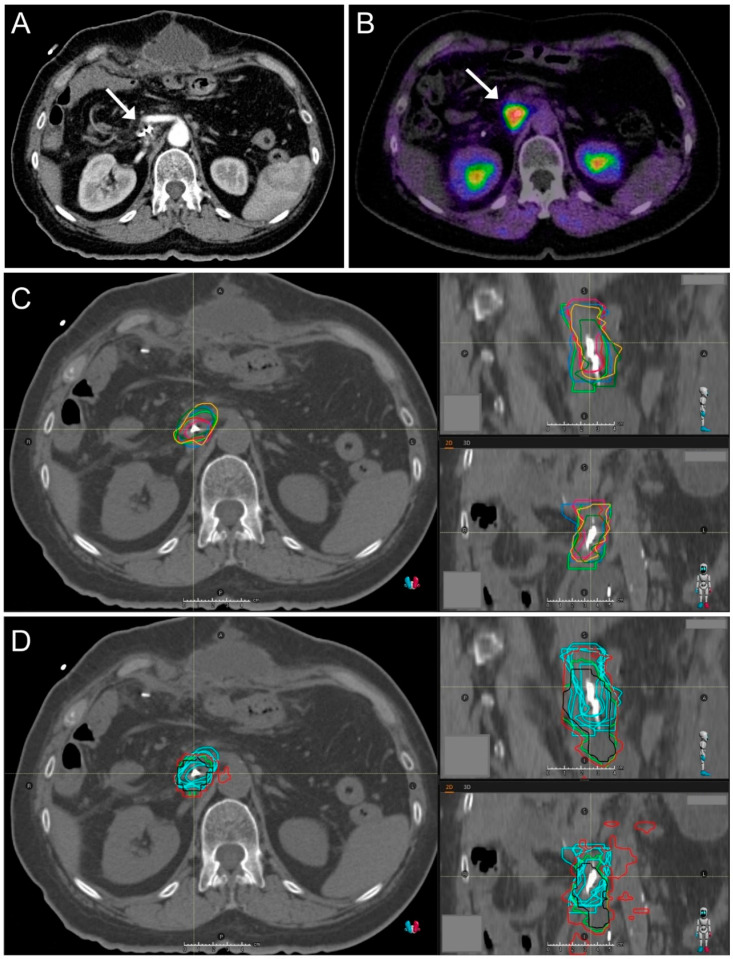
Imaging and target definition of a 66-year old patient with locally recurrent pancreatic cancer showing convincing concordance of FAPI avidity and manually defined cancer tissue. (**A**) Contrast-enhanced computed tomography (CT) scan. The local tumor recurrence is adjacent to an operation clip, as marked by the white arrow. (**B**) Fused slice of a fibroblast activation protein inhibitor positron emission tomography (FAPI-PET)/CT scan. The red area represents a high uptake of the FAPI-tracer (white arrow). (**C**) Gross tumor volumes (GTVs) of six different radiation oncologists, delineated on a conventional CT scan. An axial slice is shown on the left, sagittal and coronal slices are shown on the upper right and lower right, respectively. Each radiation oncologist’s GTV is delineated with a different color. (**D**) Automated contoured GTVs of three different FAPI thresholds (red: 1.5, green: 2.0, black: 2.5), CT slices arranged as in C. For comparison reasons, the manually defined radiation oncologist’s GTVs of C are demonstrated in light blue.

Distinct differences in GTV sizes were observed when comparing the GTVs of all radiation oncologists of each patient. In most of the cases, GTV sizes between the radiation oncologists differed up to more than 100%. The observed differences in GTV sizes are demonstrated in a violin plot in Figure 1. Precise definition of GTV was often complicated due to the relatively low Sensitivity and Specificity of CT imaging in locally recurrent pancreatic cancer (Figure 2). All patients presented with a pre-radiotherapy AJCC stage III and six of seven patients were female (P1–P6). One representative GTV definition is shown in Figure 3.

### 3.3. Interobserver Variability 

**Figure 4:**
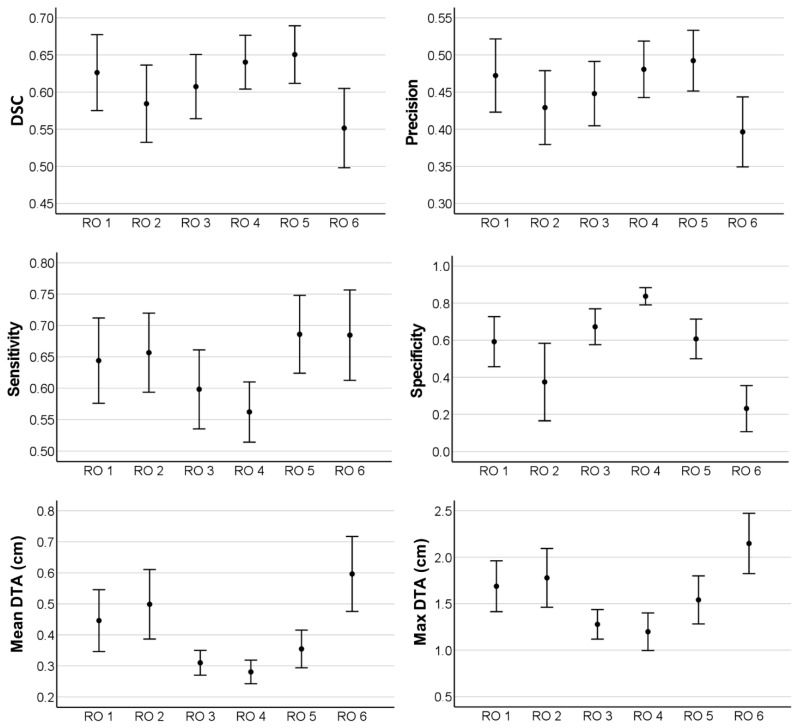
Statistically significant different volume geometries (Dice similarity coefficient: DSC, Precision, Sensitivity, Specificity, Mean distance to agreement: Mean DTA, Maximum distance to agreement: Max DTA) between six different radiation oncologists (RO1–RO6) showing high interobserver variability in gross tumor volume (GTV) definition in seven locally recurrent pancreatic cancer cases. Mean values and standard deviations are shown.

The mean DSC of the radiation oncologists differed between 0.55 and 0.65 (Figure 4). The mean observed Precision, Sensitivity, and Specificity were 0.40–0.49, 0.60–0.69, and 0.23–0.84, respectively. The mean DTA was at 0.3–0.6 cm and the max DTA was at 1.3–2.1 cm. For each analyzed volume geometry, one-way variance analysis revealed a statistically significant difference (*p* < 0.05) between the observers (DSC: *p* = 0.02; Precision: *p* = 0.02; Sensitivity: *p* = 0.03; Specificity: *p* = 0.0; Mean DTA: *p* = 0.0; Max DTA: *p* = 0.0). 

### 3.4. GTV Size Comparison

**Figure 5:**
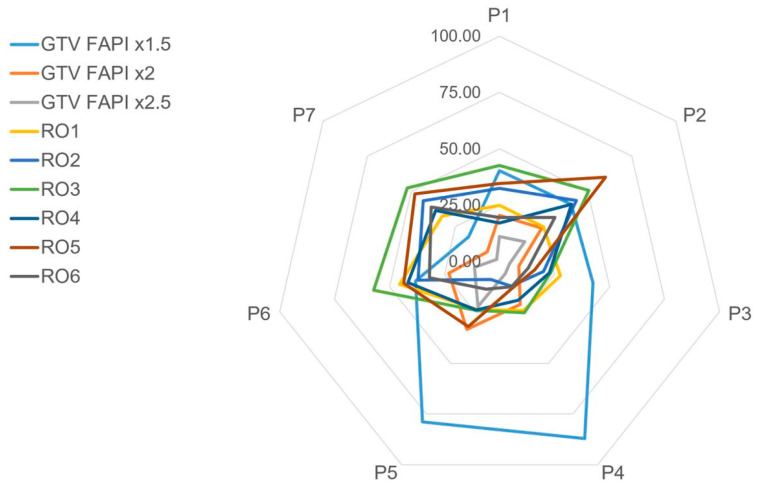
Spider plot comparing mean gross tumor volume (GTV) sizes in ccm of individual patients (P1–P7) as contoured manually using conventional computed tomography (CT) by six different radiation oncologists (RO1–RO6). In addition, 3 different automatically contoured GTVs using fibroblast activation protein inhibitor (FAPI)-PET/CT are displayed. Variations in GTV sizes of ROs can be noticed as well as the similarity of GTV FAPI x2 to RO-GTVs. The low threshold of GTV FAPI ×1.5 included several peritumoral areas in patients 4 and 5 with low FAPI-avidity (also shown in Figure 3).

The median GTVs of the radiation oncologists varied between 15.8 and 42.3 ccm (ranges from 8.9–59.5 ccm). A median of 38.5, 21.0, and 10.0 ccm could be observed for FAPI-GTVs with the thresholds 1.5, 2.0, and 2.5, respectively (ranges: 16.7–85.5 ccm, 6.0–31.0 ccm and 1.3–21.0 ccm). A comparison of GTV sizes of different radiation oncologists and several FAPI-thresholds is shown in Figure 5. Using the Wilcoxon Rank Sum Test, there was no significant difference between the volumes of automatic FAPI-GTVs based on the threshold of 2.0 and four of the six manually contoured GTVs by radiation oncologists. In a final review of all cases by board certified radiation oncologists, FAPI-GTVs based on the threshold of 2.0 were very similar to GTVs manually contoured by radiation oncologists. 

## 4. Discussion

Target volume definition of locally recurrent PDAC by six radiation oncologists based on conventional CT led to high interobserver variability in GTV sizes of up to 100%. To achieve more consistency, precision, and objectivity, we for the first time used the biological information revealed by FAPI-PET/CT to automatically delineate tumors in seven challenging, recurrent PDAC cases. The resulting GTVs were reliably generated and were comparable to the manually contoured GTVs. 

Modern radiation techniques require accurate and reliable GTV definition. However, there is a lack of Sensitivity and Specificity of CT imaging of locally recurrent pancreatic cancer [11]. As target volumes for radiation of recurrent pancreatic cancer are primarily based on CT, this results in uncertainty among radiation oncologists with high variability in target volumes. 

In our study, this inconsistency is revealed by the low DSC range of 0.55 to 0.65. A previous study of unresected pancreatic cancer cases by Caravatta et al. reported CT-based DSCs of 0.59 and 0.74 [22]. Recently, van der Veen et al. observed DSCs of 0.51 to 0.79 in head and neck cancer target definition [23]. Segmentation of elective nodal neck volumes achieved DSCs between 0.67 and 0.82 in the same study. In OAR definition, even deep learning-based segmentation could achieve DSCs of approximately 0.80, depending on the target [24,25]. Compared to these studies, the observed DSCs in the present analysis of recurrent PDAC cases seem relatively low. Additionally, analysis of variance showed significant differences between the observers for all tested geometries. Hence, our study demonstrates a large variation between the defined GTVs. This finding highlights the uncertainty among radiation oncologists and calls for new imaging modalities to improve target definition in locally recurrent pancreatic cancer. FDG-PET/CT reliably detects locally recurrent pancreatic cancer [11]. It could therefore be used as additional imaging in radiation planning. But on the other hand, especially in detection of regional lymph node metastases, FDG-PET/CT is limited [12].

Recently, significant FAPI avidity was demonstrated in pancreatic cancer patients [13]. Furthermore, FAPI-PET/CT imaging influences tumor staging and oncological treatment when compared to contrast-enhanced CT scans [15]. Therefore, we hypothesized that FAPI-PET/CT imaging could improve GTV definition in locally recurrent pancreatic cancer. Our approach of FAPI-based target definition aimed to reduce observer bias by automatically generating GTVs based on the intensity of FAPI avidity in the tumor. 

While looking at the FAPI-based target volume delineation, the novelty to note is that FAPI-PET/CT visualizes the important biological information of CAF density and FAP expression. 

We used three different FAPI thresholds for automated contouring, whereas the threshold of 2 times the background FAPI-SUV correlated best with the GTVs manually delineated by six radiation oncologists. These FAPI-GTVs were not significantly different to most of the manual GTVs when compared in size (Figure 5). Moreover, when reviewing all seven locally recurrent pancreatic cancer cases, the FAPI-GTVs matched convincingly well with the GTVs of six radiation oncologists, all experienced in the field of pancreatic radiation oncology (representatively shown in Figure 3D).

The relatively low threshold of 1.5 showed very large GTVs, consisting of the anatomically apparent tumor but also several peritumoral areas (see Figure 3D, coronal CT slice). These low-uptake lesions could be a result of unspecific background FAPI avidity or be due to peritumoral inflammation and desmoplasia [26]. Recent studies however have also suggested that CAFs provide the tumors with a pro-cancerous microenvironment and can serve as the leading structures for cancer progression [27,28]. These low-uptake lesions surrounding the tumor might therefore be precancerous, so far overlooked by radiation oncologists, thus requiring targeted radiation [29]. This hypothesis first requires validation through the gold standard of histopathological correlation in future studies. 

On the other hand, the higher FAPI-threshold of 2.5 correlated very well with the radiation oncologists’ GTVs, too. However, the corresponding FAPI-GTVs were much smaller than the manually contoured ones, since only areas with a very high FAPI avidity—hence a high CAF density—were detected. High CAF density and FAP expression have been correlated with higher grades of malignancy [27,30]. These CAF-rich areas show higher rates of tumor migration, invasion, immunosuppression, and resistance to chemotherapy, immunotherapy, and radiation [30,31]. Higher FAPI thresholds could consequently be used for delivering higher radiation doses (boost) to these areas of higher malignancy. Moreover, with advanced radiation techniques, intra-tumoral dose escalation and de-escalation based on FAPI SUVs can lead to personalized, precise, and innovative dose painting concepts. 

The automatic target volume delineation based on FAPI-PET/CT GTVs needs several considerations and has limitations. The PET/CT resolution and slice thickness is different to that of the conventional radiotherapy planning CT. Therefore, the automatic FAPI-GTVs could only be transferred to the planning CT with three possible errors. First, image registration needed to be done but patient positioning and nutritional status differed largely. Second, the FAPI-GTV-geometry became more angular through the transferring process based on the different resolution and the performed three-dimensional image registration (Figure 3D). Geometrical comparisons of the FAPI-based GTVs and the manually contoured GTVs could therefore be misleading. Third, the tumor recurrence and its adjacent structures are subject to respiratory motion and deformation. As a consequence, we decided to focus on volume size comparisons when evaluating the accuracy of FAPI-based GTVs. In addition, board certified radiation oncologists and nuclear medicine specialists compared the two GTVs with each other, since this is considered the current standard and has already been established in previous studies, such as Syed et al. [19]. The expertise of medical experts in the field of pancreatic cancer diagnostics and therapy is further required as FAPI-uptake has not yet been demonstrated to correlate undoubtfully with histologically confirmed pancreatic cancer tissue. There is an ongoing discussion of high FAPI-uptake in case of tumor-induced pancreatitis. However, this concern is less relevant in locally recurrent pancreatic cancer patients as these patients had most or all of their pancreas removed during initial surgery.

In the present study, we demonstrate that FAPI-PET/CT enables automatic GTV contouring in locally recurrent pancreatic cancer patients with convincingly well results compared to manually contoured target volumes by six experienced radiation oncologists. Based on these results, FAPI-PET/CT can be used as an additional imaging modality to improve decision-making in target definition, especially in inconclusive cases. Furthermore, the use of FAPI-PET/CT for radiotherapy planning could standardize GTV definition and therefore decrease interobserver variability. Compared to the current gold standard contrast-enhanced CT, FAPI-PET/CT seems superior due to a high tumor to background contrast. For the first time, we could demonstrate how FAPI-based automatic contouring could improve target definition in radiation oncology in pancreatic cancer.

## Figures and Tables

**Table 1:** Patient characteristics and treatment information.

Demographics and Initial Tumor Stage and Treatment		*n*	(%)
Sex			
	Male	1	(14)
	Female	6	(86)
Age at performed planning CT imaging (median, range; in years)		66 (55–77)	
Localization of initial pancreatic cancer			
	Pancreatic head	5	(72)
	Pancreatic body	1	(14)
	Pancreatic tail	1	(14)
Histology			
	Ductal adenocarcinoma	7	(100)
Grading			
	G2	6	
	G3	1	
Initial surgery			
	Whipple procedure	3	(43)
	Total pancreatectomy	3	(43)
	Distal pancreatectomy	1	(14)
Resection status			
	R1/RX	6	(86)
	R0	1	(14)
Initial AJCC * stage			
	IA	1	(14)
	IIA	1	(14)
	IIB	3	(43)
	III	2	(29)
Pre-radiotherapy AJCC * stage			
	III	7	(100)
Pre-radiotherapy TNM † stage			
	rT4 cN0 cM0	6	(86)
	rT4 cN1 cM0	1	(14)
**Radiotherapy**			
Time from resection (median, range; in months)		23 (8–50)	
Technique			
	Carbon ions, active raster-scanning, 2 beams, supine position	7	(100)
Prescribed dose			
	48 Gy (RBE) in 12 fractions (2 weeks)	7	(100)

* AJCC: American Joint Committee on Cancer. † TNM: Tumor Node Metastasis.

## Data Availability

The raw data supporting the conclusions of this article will be made available by the authors, without undue reservation.
